# Risky sexual behaviors and their associated factors within high school students from Collège Saint André in Kigali, Rwanda: An institution-based cross-sectional study

**DOI:** 10.3389/frph.2023.1029465

**Published:** 2023-03-03

**Authors:** Emmanuel Ndagijimana, Emmanuel Biracyaza, Manasse Nzayirambaho

**Affiliations:** ^1^ Department of Epidemiology and Biostatistics, School of Public Health, University of Rwanda, Kigali, Rwanda; ^2^ School of Rehabilitation, Faculty of Medicine, Université de Montréal, Montréal, QC, Canada

**Keywords:** school-based interventions, risk factors, sexual health, sexual behavior, unprotected sex/barebacking

## Abstract

**Background:**

Risky sexual behaviors (RSBs) remain public health concerns in adolescents from sub-Saharan Africa (SSA), and these practices may increase vulnerability to reproductive health problems if no early healthcare strategies are implemented. While previous studies reported that adolescents are engaged in these RSBs due to diverse influences such as the teenage stage, urbanization, and change in the environment they experience, there is a shortage of studies on RSB among adolescents in SSA. This study assessed the magnitude of RSBs and the RSB-associated factors among in-school adolescents.

**Methods:**

School-based cross-sectional study was conducted among 263 Saint Andre school students in Kigali, Rwanda, from July 3, 2020, to September 30, 2020. Systematic random sampling techniques were employed. All data were entered into Epi-Data and analyzed using SPSS version 25. Chi-square tests and multivariable logistic regression analyses were applied to determine factors associated with risky sexual behaviors. Confidence intervals (CIs) of 95% and 5% for statistical significance were maintained.

**Results:**

Of 263 participants, 109 (41%) experienced RSB in their lifetime. Among them, 66 respondents (60.55%) utilized contraceptive methods to prevent sexual and reproductive problems that can be caused by unprotected sexual intercourse. The students who experienced domestic violence had increased odds of experiencing RSB [odds ratio (OR) = 4.22; 95% CI: 1.6–11.23] than their counterparts. Those in grade 11 (OR = 2.68; 95% CI: 1.06–6.78) and grade 12 (OR = 4.39; 95% CI: 1.82–10.56) were more likely to practice RSB than those in grade 10. Alcohol users were almost more likely to experience RSB (OR = 3.9; 95% CI: 1.97–5.5) than their counterparts. Those who lived away from their biological parents had higher likelihood of experiencing RSB (OR = 2.5; 95% CI: 1.14–4.42) than those who lived with one or both parents. Students who experienced peer pressure were more likely to engage in RSB (OR = 3.9; 95% CI: 2.01–7.51) than their counterparts.

**Conclusion:**

Promoting specific intervention programs built upon the factors associated with RSB among high school students needs to be prioritized.

## Background

Most of the students from secondary schools are often aged between 11 and 24 years. This age interval, characterized by physical maturation, is considered to have sexually active people (1, 2). A report from the United Nations International Children’s Emergency Fund (UNICEF) has recently documented that adolescence is the period that constitutes three stages of age, namely, early adolescence (from 10 to 13 years old), middle adolescence (from 14 to 16 years old), and late adolescence (from 17 to 19 years old), while those aged 20–24 years are considered young adults (3). During this transitional period between childhood and adulthood, physical maturity precedes psychological and social maturity. Thus, it is in this transition that adolescents are highly exposed to risky sexual behavior (RSB) if parents do not provide early sexual health education to their children at a younger age. RSB refers to the engagement in sexual practices and sometimes unprotected sexual intercourse (4). A similar research stated several examples of RSB, such as unprotected intercourse without male or female condom use, unprotected mouth-to-genital contact, starting sexual practices at a young age, having multiple sexual partners, and having a high-risk partner. According to previous studies (5–7), these practices might increase vulnerability to reproductive health problems. RSB can also be measured across six indicators, namely, the first experience of sex was involuntary, coercive sex in the past year, reported not using a condom at sexual debut, and reported not using a condom during the last intercourse, starting sexual practices at young age, and possession of multiple sex partners. Previous studies also reported that 42.4% had more than one sexual partner and had received cash or in-kind compensation for sex in the past year. Based on this literature, all the study participants presented more than one indicator in their previous sexual practices that allowed us to conclude RSB (8, 9).

RSB has significant effects not only on the children but also on family functioning, community resilience, and the development of the country (4–6). These behaviors left the patricians of these RSBs almost maimed, with tremendous effects on biological or physiological health, mental health, and social health. Consequently, these behaviors are extreme circumstances, and there is a need for a multidisciplinary team to prevent teenagers from these behaviors and their impact (psychosocial and physiological effects) on their lifestyle.

In most high schools, the majority of the students are teenagers. Adolescence is a stage that people encounter once throughout a lifetime. It refers to a period in which they may experience RSB, which may have adverse sexual and reproductive health outcomes (4, 10, 11). Further, healthy sexual development substantially contributes to the quality of life of the sexual users or holistic personal wellbeing. However, unaware youths and adolescents may develop diverse RSBs that may put their lives in danger (4, 6, 12). In low- and middle-income countries (LMICs), the incidence of RSB, including unprotected sexual intercourse or barebacking and early sexual initiation, is increasing; however, efforts are being made by the nations to increase awareness and attenuate the prevalence of sexual reproductive problems (10). More than 50% new sexually transmitted diseases (STDs) are documented every year as a result of RSB in young adults aged less than 24 years old (13). Further, young adults also experience high-risk behaviors, including substance users (smoking, alcohol) and gender-based violence, which lead to the RSB (7, 8). In Ethiopia and many other developing countries, a high proportion of sexual reproductive health (SRH) problems such as HIV are the result of RSB, mainly in young people. In particular, research on the AIDS epidemic established that sub-Saharan Africa (SSA) accounts for 60% of all people living with HIV/AIDS due to the RSB, which has hampered the quality of life (12, 14).

It is important to note that knowledge of HIV/AIDS, sociodemographic characteristics, education of the mother, family structure, family income per month, year of education, occupation of the mother, age of the start of sexual intercourse, peer pressure, substance use, and coercion are the factors responsible for the risky sexual behaviors among the high school students (5, 15). Further, students with poor knowledge of HIV/AIDS have greater odds of engaging in risky sexual behaviors. During transition from childhood, adolescents experience rapidly changing lifestyles by being exposed to the internet and mass media that influence them to easily engage in these practices (13, 15). In addition, the empirical evidence in Botswana has reported that young people are sexually active and they often experience their first sexual encounter at an average age below 18 years, which is considered risky sexual intercourse since they are not mature (11). Similar authors stated that 90% of the boys aged 10–14 years had their first sexual experience unintentionally, while 50% of the boys did sexual practices after planning. Additionally, more than half of girls aged 15–24 years experienced their first sexual intercourse unintentionally. Unfortunately, more than half of teenage girls experience unwanted pregnancies; however, some of the adolescents who are engaged in RSB use contraceptives (16). Regarding the factors associated with the RSB, peer pressure was reported to result in school dropout among the students from primary and secondary schools (13, 17).

In Rwanda, upper-secondary education encompasses grades 10–12 (Senior 4 to Senior 6), and the students of these grades are often 15–19 years old. However, depending on various characteristics, the students may be older than 20 years. Many young adults in Rwanda are engaged in sexual behaviors that increase their risk of sexual reproductive problems such as HIV, gonorrhea, syphilis, and *Papillomavirus*, which hinder the quality of life of the affected people (11, 18). Rwanda, like many other countries, is trying to cope with the problem of sexual behavior, especially in the young population, on the same scale as drug abuse or alcohol. This is because engagement of young people in RSBs has a negative impact on the population in general and on reproductive health in particular (19, 20). Although the present literature on RSB and its risk factors is limited, we hypothesize that the prevalence of RSB is high and sociodemographic factors are related to RSB in Rwanda, as previously reported in the other SSA countries. This study determined the magnitude of RSB and the factors associated with RSB among students from secondary schools in Rwanda.

## Materials

### Study design

An institution-based cross-sectional study design was applied among the students from Collège Saint André, a secondary school in Kigali, Rwanda. Students registered for grades Seniors 4–6 were systematically selected and enrolled in the study.

### Settings and participants

The study surveyed high school students from Collège Saint André, which is one of the biggest secondary schools in the city of Kigali, Rwanda. This study used a quantitative approach to assess the level of sexual intercourse and to identify its associated factors among this target population using an individual self-administered questionnaire. The total number of younger students in the school was estimated to be 596 out of a total of 599 students.

### Sampling procedures

**Figure 1:**
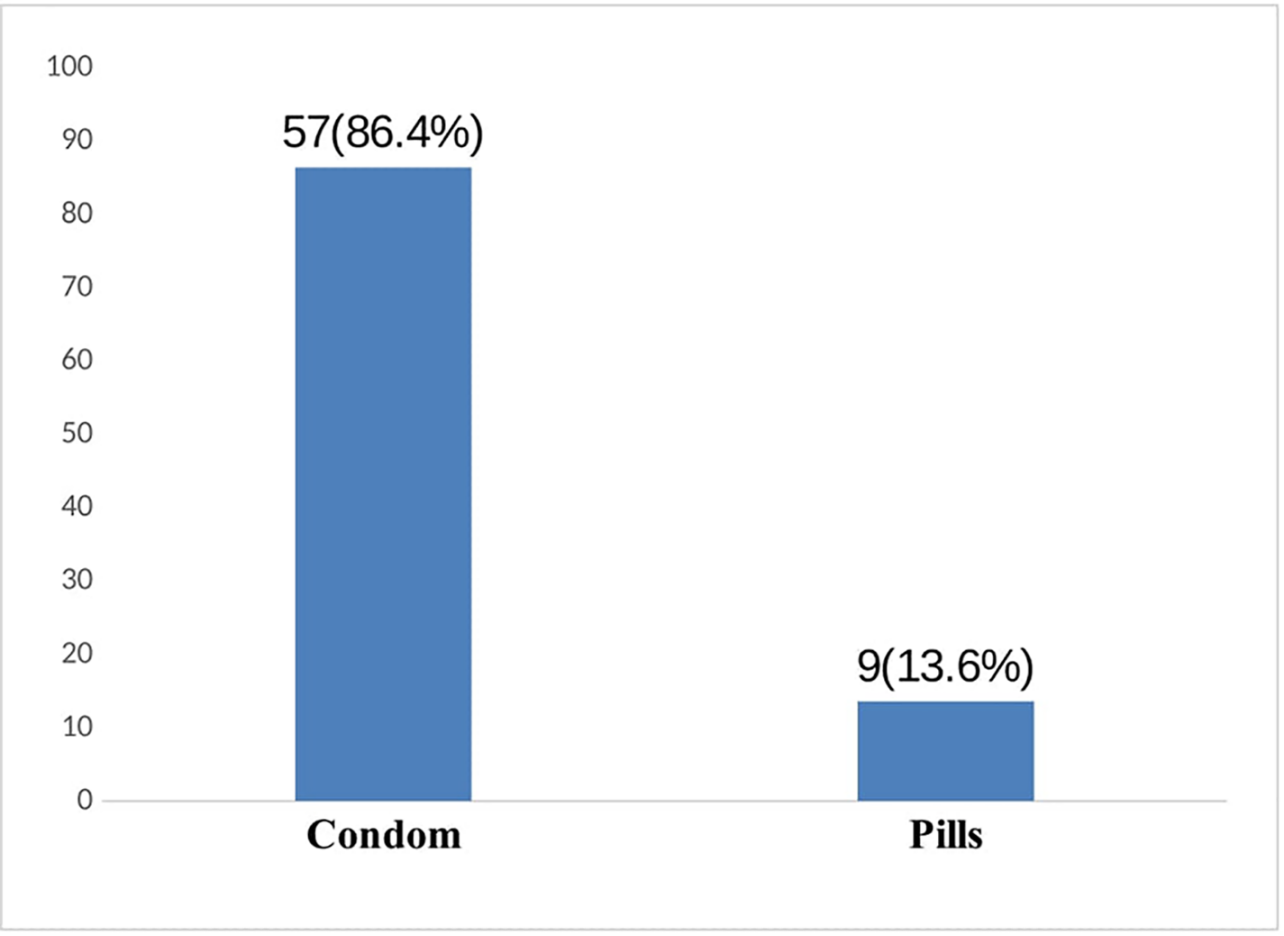
Schematic staging of sampling procedures.

A total of 596 students were found in Collège Saint André. A sampling frame containing the lists of all students under each grade level (10th, 11th, and 12th) was developed based on the lists obtained from the students’ record office in the academic year 2019 (Figure 1). Then, the sample size was calculated using a single population proportion. Formula $n=\frac{N}{1+Ne^{2}}$ was used to calculate the sample size, where “*n*” stands for the sample size, “*N*” stands for the total population, and “*e*” is the margin error (0.05%). Therefore, $n=\frac{596}{1+596{\left(0.05\right)}^{2}}$. Using this information, the calculated sample size was *n* = 239. Considering the possible nonresponses, we added 10% of extra participants; then, the total sample size of our study participants was 263. In each selected class, the sample was drawn proportionally to the size of the class (total sample size divided by the number of available students in each class). The lists of all students in each class were used to reach the participants. Each student on the list of the class was given a number. Then, all numbers were written on pieces of paper and put into a basket. The researcher picked the numbers randomly from the basket, one by one, until the calculated sample size for each class was reached. After obtaining the total sample size, the systematic random sampling technique was applied to select the interviewees.

### Study variables

The outcome variable for our study was experiencing RSB. The explanatory variables were sociodemographic factors (such as student’s age, gender, religion, and education), parental characteristics (like education of the mother and father), substance use, living arrangements, environmental variables (mode of study, peer pressure, access to channel media, or social media), and grades of the student, and communication with parents about sexual intercourse.

### Data collection

The trained graduates from undergraduate programs at the University of Rwanda performed data collection from July 3, 2020 to September 30, 2020, and all the data collectors were under the supervision of the authors. Research questionnaires were administered among the eligible participants based on their grades or classes. Indeed, before the students filled out the research questionnaires, they were informed about the research purpose. The filled questionnaires were submitted to the data collectors.

### Data analysis

Prior to statistical analyses, the collected data from the students were entered into Microsoft Excel. Double data entry was done, and the consistency of the entered data was cross-checked by comparing the two separately entered data entries on Epi-Data. Data were cleaned and entered into Epi-Data version 3.0.2 and exported to the Statistical Package for Social Sciences (SPSS) version 25 for statistical analyses. Descriptive and analytical analyses were performed. Statistical parameters such as means, frequency, and percentage were employed for descriptive analysis. In analytical analyses, bivariate logistic regression was applied to identify factors associated with RSB among the students. All significant predictors were exported to multivariate logistic regression models to obtain the significantly associated variables with respect to 95% confidence interval, and *p* < 0.05 were considered statistical significant.

### Ethics

All procedures applied in the study involving human participants were in agreement with the guidelines and regulations established in the Declaration of Helsinki 1964 and its later amendments or comparable ethical standards (21). Ethical clearance was sought from the committee of the Institutional Review Board (IRB), College of Medicine and Health Sciences of the University of Rwanda (No. CMHS/IRB/415/2019). Participants aged more than 18 years provided written consent. Students aged less than 18 years provided their consent to participate, and their parents or legal guardians provided written and signed informed consent forms. Confidentiality and voluntariness were secured and treated anonymously.

## Results

### Demographic characteristics of the respondents

The mean and standard deviation (SD) for the age of enrolled students were 17.39 and 1.11 years, respectively. The majority (185, 70.34%) of the respondents were in the age range of 17–18 years. The majority (*n *= 179, 68.1%) of the respondents were boys, and a higher number of the respondents were from grade 6 or grade 12 (*n* = 94, 35.7%). Our findings showed that most of the respondents had their mothers with university studies (*n* = 106, 40.3%) and this is similar to the education of their fathers (*n* = 103, 39.16%). More than half (*n* = 147, 55.9%) studied while spending time at school (or boarding), and only 144 respondents (43.4%) were substance users. Of 263 respondents, 117 respondents (44.5%) experienced domestic violence and 174 respondents (66.16%) were influenced by their peers to engage in sexual behaviors (Table 1).

**Table 1:** Descriptive characteristics of the students (*N* = 263).

Variable	Number	Percentage
Age of the respondent (years)		
15–16	67	25.48
17–18	185	70.34
19–20	11	4.18
Sex of the student		
Male	179	68.1
Female	84	31.9
Grade of the student		
Grade 10	88	33.5
Grade 11	81	30.8
Grade 12	94	35.7
Substance user (alcohol)		
No	149	56.6
Yes	114	43.4
Education of the mother		
No formal education	24	9.13
Primary	46	17.49
Secondary	87	33.08
University	106	40.3
Education of the father		
No formal education	37	14.07
Primary	45	17.11
Secondary	68	25.86
University	103	39.16
Domestic violence		
No	146	55.5
Yes	117	44.5
Religion		
Catholic	88	35.5
Protestant	129	49
Adventist	33	12.5
Muslim	11	4.2
Other	2	0.8
Study mode		
Boarding	147	55.9
Nonboarding	116	44.1
Living arrangements		
Living with one or both genetic parents	212	80.6
Living away from the parents	51	19.4
Ever heard about RSB		
Yes	259	98.48
No	4	1.52
Ubudehe category^a^		
Category II	37	14.07
Cetegories III and IV	226	85.93
Awareness of the effects of RSB		
Yes	217	82.5
No	16	17.5
Province of residence		
Kigali	150	57
West	16	6.1
South	36	13.7
North	25	9.5
East	35	13.7
Communicate with the parents about sexual and reproductive health		
No	86	32.7
Yes	117	67.3
Peer influence		
No	89	33.84
Yes	174	66.16
Satisfied with the school materials		
No	150	57.03
Yes	113	42.97

RSB, risky sexual behavior.

^a^ Ubudehe category: It is a Rwandan strategy of categorizing all households into one of a range of appropriate categories of the poverty level. There are four categories, as follows: Category I: very poor and vulnerable citizens who are homeless and unable to feed themselves without assistance; Category II: citizens who are able to afford some form of rented or low-class owned accommodation but who were not gainfully employed and could only afford to eat once or twice a day; Category III: citizens who are gainfully employed, even employers of laborers, small farmers who had moved beyond subsistence farming, and owners of small and medium-scale enterprises; Category IV: the citizens classified under this category were chief executive officers of big businesses, employees who had full-time employment with organizations, industries, or companies, government employees, owners of shops or markets, and owners of commercial transport vehicles or trucks.

### Proportion of sexual intercourse practices among young students

**Figure 2:**
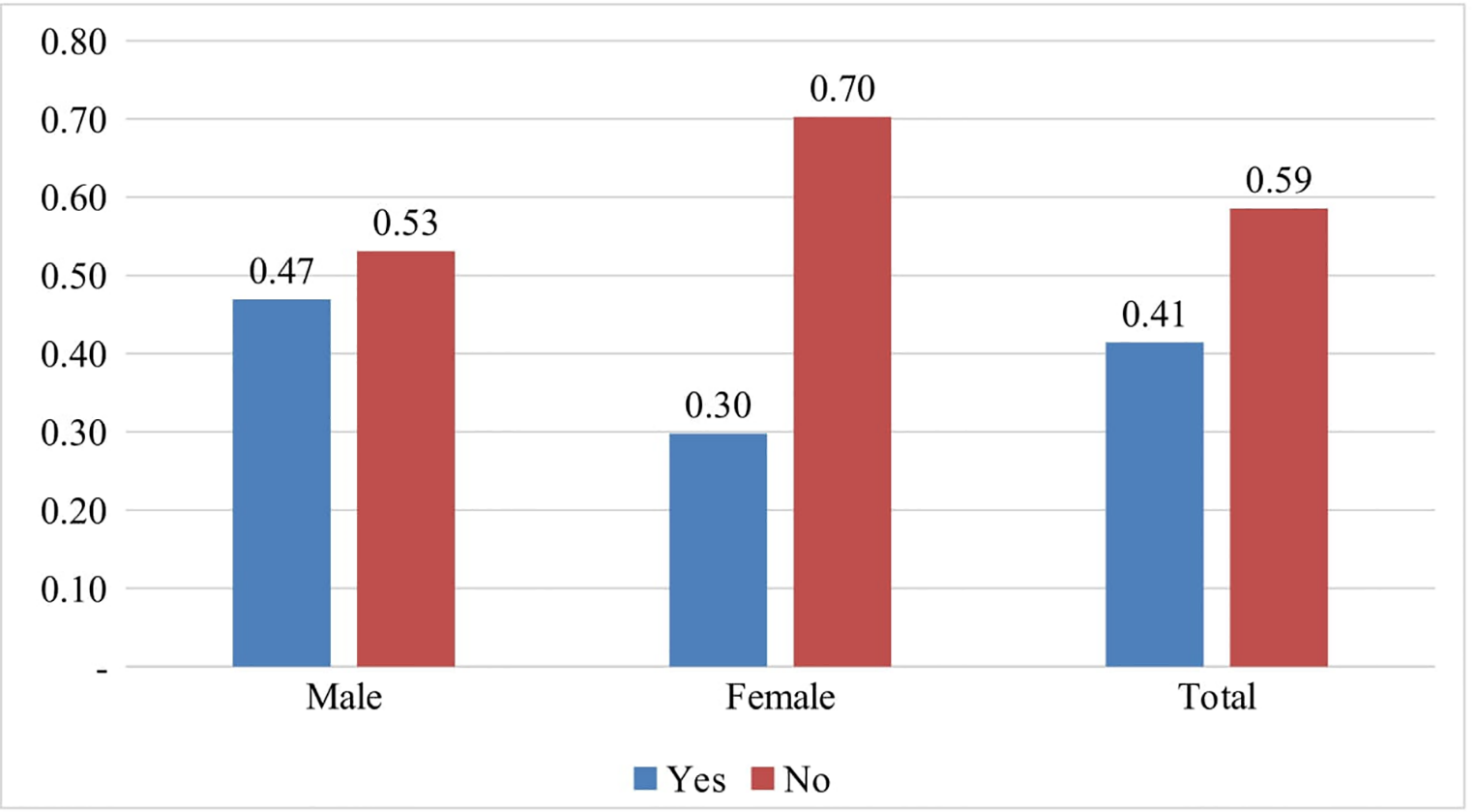
Prevalence in engagement in risky sexual behaviors among the students.

Our findings indicated that 41% (*n* = 109) of our respondents were engaged in RSB, and these behaviors could expose them to sexual and reproductive health problems. Further, the proportion of respondents engaged in RSB was 0.47 or 47% among boys and 0.3 or 30% among girls (Figure 2).

### Risky sexual behaviors among study participants from secondary schools

Our results indicated that the majority (*n* = 77, 70.64%) of the students engaged in RSB were aged 17–18 years. Most (*n* = 84, 77.06%) of the students engaged in risky sexual behaviors were boys. A higher proportion of the students engaged in risky sexual behaviors was those who resided in Kigali (*n* = 70, 64.22%). Regarding the mode of the study, our results showed that a majority (*n* = 74, *n* = 67.89%) of the respondents engaged in risky sexual behaviors were students not enrolled in boarding. The incidence of RSB was higher in substance users (*n* = 68, 79.82%) than nonsubstance users. Concerning parents’ education, our results demonstrated that the students whose mothers had secondary (*n* = 48, 44.12%) and university (*n* = 47, 43.12%) studies had a greater prevalence of experiencing RSB than the other groups. For living arrangements, high prevalence (*n* = 80, 73.39%) of risky sexual behaviors was found in those who lived with one or both parents. A higher occurrence of RSB was found in students who experienced peer influence (*n* = 89, 81.56%) than those who did not. The prevalence of experiencing risky sexual behaviors was higher in students who did not get opportunities to communicate with their parents or guardians (*n* = 78, 71.56%) than those who communicated with their parents about these behaviors. Moreover, bivariate analysis found that students having ever had sex is associated with gender, study mode, religion, study mode, living arrangements, education of the parents, peer influence, domestic violence, communication about the RSB, and substance use, and these factors were significantly associated with RSB among the students (Table 2).

**Table 2:** Proportion of risky behavior practices by demographic characteristics and predictors associated with risky sexual intercourse among students.

Variables	Engaged in risky sexual behaviors (*n* = 109)		Never engaged in risky sexual behaviors (*N* = 154)		*p*-value
	Number	%	Number	%	
Age of the student (years)					
15–16	28	25.69	39	74.31	0.340
17–18	77	70.64	108	29.36	
19–20	4	3.67	7	96.33	
Sex					
Male	84	77.06	95	22.94	0.008^a^
Female	25	22.94	59	77.06	
Residence					
Kigali City	70	64.22	80	35.78	0.238
Southern Province	14	12.84	22	87.16	
Northern Province	7	6.42	18	93.58	
Western Province	4	3.67	12	96.33	
Eastern Province	14	12.84	22	87.16	
Religion					
Catholic	40	36.70	50	63.30	0.014^a^
Protestant	51	46.79	78	53.21	
Adventist	9	8.26	24	91.74	
Muslim	9	8.26	2	91.74	
Current class of study (grades)					
Grade 10	32	29.36	53	34.42	
Grade 11	41	37.61	66	42.86	
Grade 12	36	33.03	35	22.73	
Study mode					
Boarding	35	32.11	103	67.89	<0.001^b^
Nonboarding	74	67.89	51	32.11	
Ubudehe category					
Category II	7	6.42	30	19.48	0.018^a^
Categories III and IV	102	93.58	124	80.52	
Satisfied with needs					
Yes	87	79.82	120	20.18	0.712
No	22	20.18	34	79.82	
Substance use (alcohol, smoking)					
No	68	62.39	82	37.61	0.02^a^
Yes	41	37.61	72	62.39	
Living arrangements					
Living with biological parents	80	73.39	132	26.61	0.013^a^
Away from the parents	29	26.61	22	73.39	
Level of education of the mother					
No formal education	5	4.59	19	95.41	<0.001^b^
Primary	9	8.26	37	91.74	
Secondary	48	44.04	39	55.96	
University	47	43.12	59	56.88	
Education of the father					
No formal education	12	11.01	35	88.99	<0.023^a^
Primary	55	50.46	48	49.54	
Secondary	24	22.02	21	77.98	
University	18	16.51	50	83.49	
Ever heard about sexuality					
Yes	107	98.17	152	1.83	0.726
No	2	1.83	2	98.17	
Domestic violence	** **	0.00	** **	100.00	** **
No	47	43.12	89	56.88	<0.001^b^
Yes	62	56.88	55	43.12	
Know the possible consequences of risky sexual intercourse					
Yes	99	90.83	148	9.17	0.078
No	10	9.17	6	90.83	
Peer influences					
No	20	18.35	59	81.65	0.004^a^
Yes	89	81.65	85	18.35	
Communicate with the parents about RSB					
No	78	71.56	119	28.44	0.027^a^
Yes	31	28.44	35	71.56	

RSB, risky sexual behavior.

^a^ *p* < 0.05.

^b^ *p* < 0.001.

### Multivariate logistic regression models for assessing the factors associated with RSB among students of secondary school

In multivariable logistic regression analyses, the gender of the student, year of education, domestic violence, type of schooling, parental education, drinking alcohol, religion, peer pressure, lack of communication between parents and children about sexual issues, and poor parental monitoring were factors significantly associated with RSB among secondary school students in the study area. Girls were roughly four times more likely to engage in risky sexual behaviors [odds ratio (OR) = 3.73; 95% confidence interval (CI): 1.6–7.84] than boys. Respondents who experienced domestic violence had greater odds to experience RSB (OR = 4.22; 95% CI: 1.6–11.23) than their counterparts. Those in grade 11 (OR = 2.68; 95% CI: 1.06–6.78) and grade 12 (OR = 4.39; 95% CI: 1.82–10.56) were more likely to encounter RSB than those in grade 10. The likelihood of being at RSB among female students who consumed alcohol was seven times higher than those who did not consume alcohol (OR = 3.9; 95% CI: 1.97–5.5). Regarding religion, Muslims were less likely to experience RSB (OR = 0.09; 95% CI: 0.01–0.63) than Catholics. Those whose fathers had formal education were less likely to engage in RSB (OR = 0.54; 95% CI: 0.3–0.97) than those whose fathers had no formal education. Also, those whose fathers had primary education were less likely to experience RSB (OR = 0.12; 95% CI: 0.1–0.31) than those fathers no formal education. Students whose mothers had secondary education were five times more likely to experience RSB (OR = 5.1, 95% CI: 1.54–16.8) than those whose mothers had no formal education. Further, students who lived away from their parents had more likelihood of experiencing RSB (OR = 2.5; 95% CI: 1.14–4.42) than those living with one or both genetic parents. Those who were under peer pressure influence were about four times more likely to engage in RSB (OR = 3.9; 95% CI: 2.01–7.51) than their counterparts. The students who did not have opportunities to communicate on the SRH issues with their parents or guardians in the last year were more likely to engage in RSB (OR = 0.81; 95% CI: 0.7–0.98) than their counterparts (Table 3).

**Table 3:** Factors associated with RSB among sexually active school students in Kigali, Rwanda.

Variables	OR	95% confidence intervals		*p*-value
		Lower bound	Upper bound	
Class level				
Grade 10	1			.004^b^
Grade 11	2.68	1.06	6.78	0.04^a^
Grade 12	4.39	1.82	10.56	<0.001^c^
Sex of the student				
Male	1			
Female	3.73	1.6	7.84	<.001^c^
Religion				
Catholic	1			0.10
Protestant	0.18	0.03	1.15	0.07
Adventist	0.16	0.03	0.97	0.05
Muslim	0.09	0.01	0.63	0.02^a^
Mode of study or schooling				
Boarding	1			
Not boarding	1.38	1.12	1.69	<0.001^c^
Education of the mother				
No formal education	1			0.01^a^
Primary	0.5	0.41	0.63	<0.001^c^
Secondary	0.43	0.2	0.95	0.037^a^
University	0.29	0.16	0.5	<0.01^b^
Education of the father				
No formal education	1			
Primary	0.54	0.3	0.97	0.02^a^
Secondary	0.36	0.17	0.74	0.007^a^
University	0.12	0.1	0.31	<0.001^c^
Substance users (alcohol and tobacco)				
No	1			
Yes	3.9	1.97	5.5	<0.001^c^
Domestic violence				
No	1			
Yes	4.22	1.6	11.23	<0.01^b^
Living arrangements				
Living with one or both parents	1			
Living away from the parents	2.5	1.14	5.42	0.023^a^
Ubudehe category				
Category II	1			
Categories III and IV	2.14	1.01	4.53	0.047^a^
Peer influence				
No	1			
Yes	3.9	2.01	7.51	<0.001^c^
Communicate with the parents or guardians about the risky sexual behaviors				
No	1	** **	** **	** **
Yes	0.81	0.7	0.98	0.028^a^

AOR, adjusted odds ratio.

^a^ *p* < .05.

^b^ *p* < 0.01.

^c^ *p* < 0.001.

## Discussion

This study determined the prevalence of RSB and its associated factors among high school students from College Saint André. Our results revealed that 41% of respondents were engaged in RSB. Our results are in agreement with the previous studies conducted in SSA countries, such as Ethiopia and Kenya, where the prevalence varies between 40% and 50% (22). Among the students with risky sexual behaviors, 39.45% had unprotected sexual intercourse. These results collaborated with previous studies that the students exposed to risky sexual behaviors do not use contraceptive methods, which may lead to sexual and reproductive health problems (23, 24).

Moreover, girls were more likely to experience RSB in secondary schools. Our results are in harmony with the previous studies conducted in Tanzania, Nigeria, and South Africa and in contrast to the result of a study conducted in Ethiopia (12, 16, 25). Interestingly, the students who faced peer pressure are more likely experience RSB, which is in line with previous studies in Ethiopia, South Africa, and Assela (7, 12, 26), and this might be due to their need to share life experiences with their peers. These results contradicted the previous study carried out in Addis Ababa that documented no significant association with peer influences, which might be due to the self-efficacy of the students toward external forces (27). In congruence with previous studies (28), another notable finding of our study is that substance users were more prone to experience risky sexual behaviors than their counterparts. These behaviors associated with RSB might put young people at risk for HIV, STDs, and early pregnancies (in girls). These findings are similar to those of previous studies conducted in Thailand, Kenya, Ethiopia, Nigeria, and Tanzania (26, 29–31).

Our results indicated that the students whose families are in the third Ubudehe category had greater odds of experiencing risky sexual behaviors. This was because the adolescents from the third category are children of citizens who were gainfully employed, employers of laborers, small farmers with subsistence farming, or owners of small- and medium-scale businesses. Our results are in collaboration with prior studies that conveyed that students from rich families had greater risks of experiencing risky sexual behaviors (24, 32). Our results also conflict with prior studies that conveyed that students from wealthy families have fewer odds of experiencing risky sexual behaviors (7, 27). Thus, material satisfaction and the economic class of the family contribute significantly to RSB among young students. These findings are relevant to prior studies that reported that poverty and economic depravity, in particular, are the risk factors for RSB and premarital sexual practices among young people, especially girls (23, 33). In agreement with previous studies conducted in SSA countries that indicated students living with their parents and experiencing domestic violence had a greater risk of being engaged in RSB than their counterparts (6, 12, 34), our study revealed that living without biological parents or experiencing domestic violence are associated with an increased prevalence of RSB. This was because the students who do not live with their genetic parents also do not get sexual and reproductive health education and easily experience a lack of coparenting and parental monitoring. Practicing RSB for the studies might be due to experiencing a lack of parenting or poor parenting or parental monitoring as well as psychosocial effects associated with domestic violence and the absence of the parents (35, 36).

In addition, the students who experienced peer pressure were more likely to have had sexual intercourse than their counterparts. Our results are consistent with preceding studies (12, 28, 37). The students whose parents had high education and university studies were less likely to experience RSB than their counterparts. These results are supported by previous studies (9, 12, 31). Besides, the nonboarding students were more engaged in RSB than the boarding students. Our results challenged previous studies that agreed that boarding students were more engaged in RSB than nonresidents because the boarding school environment provides greater freedom and, consequently, perhaps more permissive sexual attitude (38). The reason for our results is that the school has school leaders who are in charge of discipline and they effectively communicate with the parents and guardians to improve the behaviors of the students.

### Limitations and future directions

Although this study expands and builds on the current literature with the associated factors of RSB and its impact on young adults, the authors encountered several shortcomings that need to be further considered. The study had a small sample size where it targeted only students from one secondary school, which may not be generalized to all students of all national schools. Indeed, the study was limited to participants from a secondary school from an urban area and may not be representative of all young adults out of school in the area that need attention when generalizing the findings to other young adults, particularly teenagers. Second, as the findings from the present study are based on the cross-sectional data, causal relationships could not be inferred and the results should be taken with caution. It is in that regard the longitudinal study design is recommended to determine the causal relationships. Additional work using a large sample is critical to investigating similar studies using qualitative methods with the aim to understand their perspectives on risky sexual practices. Further, we recommend the concurrent and longitudinal studies to survey the medication of sociodemographic and psychosocial factors of RSB using the longitudinal study design. A notable strength of the present study is that unlike previous studies it was conducted in a school; however, the previous explored the sexual reproductive health of adolescents.

## Conclusion

Our study documented the considerable prevalence of RSB among young students. The lack of parental monitoring, lack of discussion between youth and their parents, religion, peer pressure, substance use, parental education, mode of study, access to media channels, gender of the child, and performance in the class were significantly associated with RSB among secondary school students. Discussion between parents or caregivers and children on sexual reproductive health and sexual practices and their harmful influence is substantial. We recommend that sexual risk prevention programmes for adolescents be designed and implemented in educational institutions; additionally, it is critical to strengthen family education and sexual behaviour education beginning in primary school. This is to equip youths with correct information to enable them make informed choices about responsible sexual life. There is a need to promote specific intervention programs built upon those factors that are associated with an increased likelihood for early sexual introduction and risky sexual behavior. Parents are recommended to educate their children about SRH when they are too young, and this can enrich their knowledge about sexual health and enhance their informed decision about SRH issues. As a result, our findings suggest that psychological and behavioral programs for young people be reinforced in order to address RSK behaviors.

## Data Availability

The original contributions presented in the study are included in the article/Supplementary Material, further inquiries can be directed to the corresponding author.
